# Socio-economic inequity in accessing malaria control interventions in Nigeria: analysis of changes between 2003 and 2008

**DOI:** 10.1186/1475-2875-11-S1-P6

**Published:** 2012-10-15

**Authors:** AbdulGafar Alawode, Olalekan A Uthman, Ismail Yahaya

**Affiliations:** 1 Partnership for Transforming Health System II, Abuja, Nigeria; 2 Division of Epidemiology, institute of Environmental Health, Karolinska Institutet, Stockholm, Sweden; 3 Department of Public Health Sciences, Mid-Sweden University, Sundsvall, Sweden

## Background

Malaria is the major health problem in Nigeria, accounting for 60% of outpatient consultations and 30% of hospital admission [1]. The Federal Ministry of Health (FMoH) of Nigeria has adopted cost-effective malaria control interventions as tools for achieving ambitious objective of halving malaria burden by 2013 as contained in the National Malaria Strategic Plan (NMSP). The interventions include prompt and effective case management, Insecticide Treated Net (ITN) and Intermittent Preventive Treatment (IPT) while parasitological diagnosis is an adjunct to effective case management. The coverage for these interventions is very low as only 8% of households own ITN, 1.1% of under five children with malaria have access to Artemisinin-based Combination Therapy (ACT) within 24 hours of onset of fever and only 6.5% of pregnant women have access to 2 doses of IPT [2].

## Methods

We used concentration index (*C*) to measure changes in socioeconomic inequality in access to malaria control measures using Nigeria Demographic and Health Surveys 2003 and 2008.

## Results

There was increase in access to all the malaria control measures studied between 2003 and 2008: ownership of any bed nets (11.8% to 16.9%), under-5 children who slept under treated bed net (1.4% to 5.3%), under-5 children with fever who received non-Artemisinin-Combination Therapies (ACT) (9.6% to 27.5%) and pregnant women who received intermittent preventive treatment (1.1% to 7.8%). In 2008 there is concentration of treated net use among the rich (pro-rich inequality) which was more pronounced in the North West and South east regions and least pronounced in the South South. The pattern of inequalities of use treated bed nets were similar those observed in ownership of treated net. There is pro-rich inequality in the prompt and effective treatment of malaria using non-ACT, ACT combination and use of intermittent preventive treatment by pregnant women. In most cases the inequalities were more pronounced in the northern regions.

## Conclusion

Though access to most malaria control interventions increased across all wealth quintile between 2003 and 2008, there are significant differences in access to some of these interventions that favour the better-off of society as a whole and some geopolitical regions.

## References

[B1] Federal Ministry of Health Nigeria Nigeria. National Malaria Strategic Plan: 2009 - 2013 2009 Abuja, Nigeria: FMoH, Nigeria

[B2] National Population Commission (NPC) [Nigeria] ICF Macro Nigeria Demographic and Health Survey 2008 Abuja: NPC Nigeria and ICF Macro

